# Supplementary material for: House Dust Mite Induces Bone Marrow IL-33-Responsive ILC2s and TH Cells
Source: Int J Mol Sci. 2020 May 26;21(11):3751. doi: 10.3390/ijms21113751 (PMC7312993; doi:10.3390/ijms21113751)
Supplement: Supplementary file 1 [file ijms-21-03751-s001.zip › Figure S1_figure legend only.docx]

**Figure S1.** Gating strategies used in the study for **(A)** eosinophil progenitors, **(B)** mature eosinophils, **(C)** ILC2s and **(D)** T_H_ cells. EoP = eosinophil progenitors. Mat Eos = mature eosinophils. ST2 = IL-33 receptor CD125 = IL5Rα^+^. FMO = fluorescence minus one.
