# Supplementary material for: House Dust Mite Induces Bone Marrow IL-33-Responsive ILC2s and TH Cells
Source: Int J Mol Sci. 2020 May 26;21(11):3751. doi: 10.3390/ijms21113751 (PMC7312993; doi:10.3390/ijms21113751)
Supplement: Supplementary file 1 [file ijms-21-03751-s001.zip › Table S1.pdf]

Supplementary table 1. Antibodies used in flow cytometry

Antigen (clone)	Format	Manufacturer	Dilution
**Hematopoietic Lineage cocktail: CD3 (17A2), CD45R/B220 (RA3-6B2), CD11b (M1/70), TER-119 (TER-119), Ly-G6/Gr-1 (RB6-8C5)**	FITC	ThermoFisher Scientific	20 µl/test
**CD11c (N418)**	FITC	ThermoFisher Scientific	0.05 µg/test
**CD19 (1D3)**	FITC	BD Bioscience	0.25 µg/test
**NK-1.1 (PK136)**	FITC	BD Bioscience	0.5 µg/test
**FceR1 (MAR-1)**	FITC	ThermoFisher Scientific	0.125 µg/test
**CD34 (RAM34)**	FITC	BD Bioscience	0.5 µg/test
**CD3 (145-2C11)**	FITC	BD Bioscience	0.5 µg/test
**CD127/IL-7Ra (A7R34)**	PE	ThermoFisher Scientific	0.25 µg/test
**CD125 (T21)**	PE	BD Bioscience	1 µg/test
**CD4 (H129.19)**	PE	BD Bioscience	0.5 µg/test
**CD45 (30-F11)**	PerCP	BD Bioscience	0.2 µg/test
**ST2/IL-33R (RMST2-2)**	APC	ThermoFisher Scientific	0.25 µg/test
**Siglec-F (E50-2440)**	AF647	BD Bioscience	0.2 µg/test
**CD25/IL-2RA (PC61)**	BV421	BD Bioscience	0.2 µg/test
**CD193/CCR3 (J073E5)**	BV421	BioLegend	0.25 µg/test
**IL-5 (TRFK5)**	BV421	BioLegend	0.25 µg/test
**Isotype control IL-5/Rat IgG1, κ (RTK2071)**	BV421	BioLegend	0.25 µg/test
